# Comparison between Single-Dose Esomeprazole- and Pantoprazole-Based Triple Therapy on the Effectiveness for *Helicobacter pylori* Eradication in Taiwanese Population

**DOI:** 10.1155/2012/674324

**Published:** 2012-07-04

**Authors:** Hsiang-Yao Shih, Sophie S. W. Wang, Chao-Hung Kuo, Fu-Chen Kuo, Yi-Yu Chen, Meng-Chieh Wu, Bi-Chuang Weng, Yi-Chern Lee, Chi-Tan Hu, Deng-Chyang Wu, Yen-Hsu Chen

**Affiliations:** ^1^Division of Gastroenterology, Department of Internal Medicine, Kaohsiung Medical University Hospital, Kaohsiung, Taiwan; ^2^Department of Internal Medicine, Pingtung Hospital, Department of Health, Pingtung, Taiwan; ^3^Cancer Center, Kaohsiung Medical University Hospital, Kaohsiung, Taiwan; ^4^Department of Medicine, Faculty of Medicine, College of Medicine, Kaohsiung Medical University, Kaohsiung, Taiwan; ^5^Department of Gynecology and Obstetrics, E-Da Hospital, I-Shou University, Kaohsiung, Taiwan; ^6^Division of Gastroenterology, Department of Internal Medicine, Buddhist Tzu Chi Hospital and Tzu Chi University, Hualien, Taiwan; ^7^Division of Infectious Diseases, Department of Internal Medicine, Kaohsiung Medical University Hospital, Kaohsiung 807, Taiwan

## Abstract

*Background and Study Aims.* To compare the effectiveness of two regimens, single-dose esomeprazole- and pantoprazole-based triple therapy, for *Helicobacter pylori* (*H. pylori*) eradication. *Patients and Methods.* A total of 453 patients were enrolled for *H. pylori* eradication. They were randomly assigned to either EAC group (Esomeprazole 40 mg once daily, Amoxicillin 1 g twice daily, Clarithromycin 500 mg twice daily for 7 days) or PAC group (Pantoprazole 40 mg twice daily, Amoxicillin 1 g twice daily, Clarithromycin 500 mg twice daily for 7 days). Follow-up endoscopy or urea breath test was scheduled 12–16 weeks after the eradication to evaluate the therapeutic response. *Results.* Higher eradication rate in EAC group than PAC group was shown by intention-to-treat analysis (EAC 72% versus PAC 55%, *P* < 0.05) and per-protocol analysis (EAC 91% versus PAC 72%, *P* < 0.05). The incidence of adverse effects (EAC 19% versus PAC 17%, *P* = 0.712) and the compliance (EAC 87% versus PAC 91%, *P* = 0.083) were comparable between these 2 groups. *Conclusions.* Single-dose esomeprazole-based triple therapy is effective for *H. pylori* eradication.

## 1. Introduction

Chronic *Helicobacter pylori* (*H. pylori*) infection is responsible for gastritis, peptic ulcer disease, gastric mucosa-associated lymphoid tissue lymphoma (MALT lymphoma) [1], and gastric adenocarcinoma [2]. Consequently, eradication of *H. pylori* is indicated for patients with peptic ulcer disease, low-grade gastric MALT lymphoma, atrophic gastritis. First-degree relatives of gastric cancer patients and some extraintestinal diseases, for example, unexplained iron deficiency anemia, and chronic idiopathic thrombocytopenic purpura may benefit from *H. pylori* eradication as well [2]. According to the Maastricht III Consensus Report, the recommended first-line treatment of *H. pylori* eradication is triple therapy with a proton pump inhibitor (PPI), clarithromycin, and amoxicillin or metronidazole given twice daily [2].

Proton pump inhibitor (PPI) is superior to H2 blocker for *H. pylori* eradication [3] because PPI is the most potent drug to inhibit gastric secretion to enhance the bioavailability of the antibiotics in the stomach [4]. PPI is metabolized via hepatic enzyme cytochrome P450 system, especially S-mephenytoin 4′-hydroxylase (CYP 2C19) and CYP 3A4 [5]. Single-nucleotide polymorphism (SNP) of these enzymes may lead to variable plasma level of PPI and affect intragastric pH level as a result. Esomeprazole is the *S*-enantiomer of omeprazole. This single enantiomer is shown to be more efficacious than the racemic mixture of omeprazole. Although esomeprazole and its metabolites are indistinguishable from omeprazole, a single oral dose of 40 mg esomeprazole generally results in peak plasma esomeprazole concentrations of 0.5–1.0 mg/L within 1–4 hours [6]. Theoretically, esomeprazole (40 mg once daily) should be as effective and economic for *H. pylori *eradication as the regular bid dose of PPI, suggested by Maastricht III consensus. Although some studies showed the effectiveness of esomeprazole-based triple therapy for *H. pylori* eradication, they studied esomeprazole 40 mg twice daily [7, 8], instead of esomeprazole 40 mg once daily. Therefore, we conducted the study to evaluate the effectiveness of single-dose 40 mg once daily esomeprazole based triple therapy for *H. pylori* eradication.

## 2. Patients and Methods

### 2.1. Patients and Study Design

A total of 501 dyspeptic patients were included and 453 patients (192 men and 261 women, mean age 52.48 years old, 16–83 years old) were enrolled at the Outpatient Department of the Division of Gastroenterology, Kaohsiung Medical University Hospital, Kaohsiung, Taiwan, from March 2005 to March 2009. Exclusion criteria were recent use of antibiotics, bismuth, or PPIs within the prior 4 weeks; history of gastric surgery; allergy to the study medication; serious comorbid illness (decompensated liver cirrhosis, renal failure); women who are pregnant and breastfeeding; previous *H. pylori *eradicated therapy. All of them received esophagogastroduodenoscopy (EGD). In addition, all of the patients were interviewed by a trained interviewer for the personal and medical history obtained by a standardized questionnaire. Once the status of *H. pylori *infection was confirmed, participants were randomly assigned to two groups: EAC group (esomeprazole 40 mg once daily, amoxicillin 1 g twice daily, clarithromycin 500 mg twice daily for 7 days) or PAC group (pantoprazole 40 mg twice daily, amoxicillin 1 g twice daily, clarithromycin 500 g twice daily for 7 days). Follow-up endoscopy or urea breath test was scheduled 12–16 weeks after the eradication to evaluate the therapeutic response and PPI was withheld 2 weeks beforehand. This study was approved by Institutional Review Board and Ethical committee of Kaohsiung Medical University Hospital and we obtained written informed consents from all the participants.

### 2.2. Questionnaire

The standardized questionnaire consisted of demographic data, underlying diseases, use of nonsteroidal anti-inflammatory drug (NSAID) and personal history about smoking and alcohol, coffee, or tea drinking. Smokers were defined as consumption of more than one pack of cigarettes per week. Drinkers were defined as consumption of more than one glass of alcoholic beverage per day. Compliance was defined as good (taking more than 70% of all administered medication) and poor [9]. The adverse events included diarrhea, constipation, abdominal pain, anorexia, nausea, vomiting, skin rash, headache, dizziness, taste perversion, and fatigue. The adverse events were further divided into positive adverse events defined as those who considered the adverse events disturbing the quality of daily life and negative ones defined as those who did not experience the events or did not consider them troublesome [9].

### 2.3. Diagnosis of **H. pylori ** Infection

Culture, histology, rapid urease test, and ^13^C-urea breath test (UBT) were used in this study. Endoscopic biopsy specimens were rubbed on the surface of a Columbia blood agar plate for culture. Positive culture was considered if one or more colonies showed Gram negative, oxidase(+), catalase(+), urease(+), or spiral or curved rods in morphology. The presence of* H. pylori* in the pathology of gastric biopsy specimens was also evaluated by experienced pathologists. The result of rapid urease test (sensitivity 93–97%, specificity 98%) [10], CLO test (Delta West Bentley, WA, Australia), was interpreted as positive if the color turned to pink or red at room temperature 6 hours after the EGD examination. The ^13^C-urea breath test used in the study was manufactured by the Institute of Nuclear Energy Research, Taiwan. *H. pylori* infection was defined as positive either culture was positive or at least two positive results of rapid urease test, histology, or UBT [11].

### 2.4. Statistical Analysis

The primary outcomes were rates of eradication, adverse events, and compliance. The difference of the age of the patients was analyzed by Student's *t*-test. The eradication rate, adverse effects and compliance between EAC and PAC groups were analyzed by Chi-square test. *P *value < .05 was considered statistically significant.

## 3. Results 

### 3.1. Demographic Characteristics

**Figure 1:**
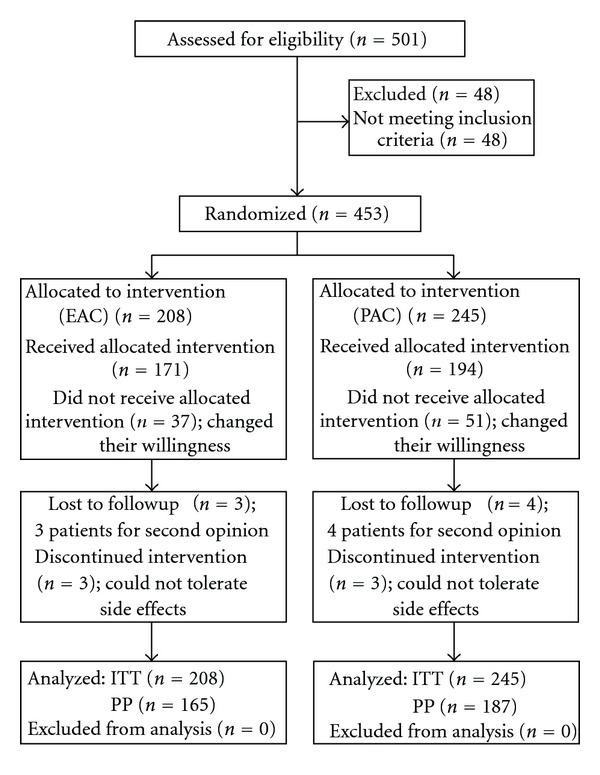


The demographic characteristics, including age, gender, smoking, alcohol consumption, ingestion of coffee or tea or both and no significant difference demonstrated, and endoscopic diagnosis of both groups (EAC group and PAC group) were analyzed (Table 1). No significant difference was found between the two groups except age and alcohol consumption (Table 1). The patient disposition according to CONSORT statement was shown (Figure 1) [12].

### 3.2. Eradication Rate

The eradication rate of *H. pylori* between the two groups was shown in Table 2. The eradication rate in the EAC group was significantly better than the PAC group in both the intention-to-treat (ITT) and the per-protocol (PP) analyses.

### 3.3. Adverse Events and Compliance

There was no difference regarding adverse effects during the treatment (EAC versus PAC, 19% versus 17%) (Table 2). In our study, adverse events included abdominal symptoms (diarrhea, constipation, abdominal pain, nausea, vomiting), taste perversion, anorexia, dizziness, headache, fatigue, and skin rash. Of all the adverse events taste perversion (EAC group 32 patients (15.4%); PAC group 29 patients (11.9%)) was the most common, followed by dizziness (EAC group 12 patients (5.8%); PAC group 11 patients (4.5%)). Fatigue (EAC 4.8%) and diarrhea (PAC 4.1%) also topped the list (Table 3). As for the compliance, 87% in the EAC group and 91% in the PAC group were noted. No significant difference was noted.

## 4. Discussion 

Our study demonstrated higher eradication rate of *H. pylori* with single-dose esomeprazole based triple therapy (esomeprazole 40 mg once daily, amoxicillin 1 g twice daily, clarithromycin 500 mg twice daily for 7 days) than pantoprazole-based triple therapy (pantoprazole 40 mg twice daily, amoxicillin 1 g twice daily, clarithromycin 500 mg twice daily for 7 days). Similar prevalence of adverse events and compliance were observed between the two groups. Proton pump inhibitors (PPIs) are primarily metabolized via hepatic cytochrome P450(CYP)2C19 pathway. Genetic polymorphisms in CYP2C19 has been shown to have great influence on the metabolism of the PPIs. In our study, esomeprazole, s-isomer-omeprazole, is less influenced than pantoprazole. Consequently, it is more likely that esomeprazole may keep its therapeutic potency persistently [13–16]. 

Another issue which matters with the potency of PPI is to tackle the increasing antibiotic resistance. Increasing prevalence of resistant strain of *H. pylori* to clarithromycin was demonstrated in some studies. According to Vakil the prevalence of clarithromycin-resistant strain in the United States was 10–12% and wider range of 1–21% in the Europe. In Asia, a study from Hong Kong disclosed that the prevalence was 7.8% and the prevalence in Taiwan was 6% [17–19]. PPI could enhance the bioavailability and activity of the clarithromycin by reducing gastric acid secretion. In other words, the more potent the PPI is, the more effective clarithromycin would be. Esomeprazole gets more anti-*H. pylori* activity by its potent suppression of gastric acid secretion. This may be an explanation why higher eradication rate of *H. pylori* was observed in the EAC group. The other explanation for the higher eradication rate in the EAC group is higher pKa1 and pKa2 values of esomeprazole. The PPI pharmacophore is a 2-pyridylmethyl-sulfinyl-benzimidazole. The differences of the structure of the current marketed PPIs (omeprazole, esomeprazole, lansoprazole, pantoprazole, rabeprazole) are about the substituents placed on the pyridine and benzimidazole rings. pKa1 means the pKa value of the pyridine nitrogen (the pH at which the number of inactive not protonated forms and that of active protonated forms are equal, in other words the relative acidic stability) and pKa2 is the pKa value of the benzimidazole N3. They two are crucial for the activation of the PPI and higher values are positively related to more potent and persistent effects [16, 20]. As reported by Roche et al., pKa1 and pKa2 values of esomeprazole are 4.06 and 0.79, respectively, and the values of pantoprazole are 3.83 and 0.11 [20, 21]. In addition, reports showed younger age and alcohol consumption had positive effects on *H. pylori* eradication [22, 23]. We also observed a similar correlation in our study. In the EAC group, which had a higher eradication rate, patients tended to be younger and have more frequent alcohol consumption. Therefore, we suggest that age, alcohol consumption and prescribed PPI are the clinical factors which may influence the eradication rate (Table 4). In conclusion the higher eradication rate observed in the EAC group was the accumulative results from more potency of esomeprazole, higher pKa1 and pKa2 values, less influence by genetic polymorphisms in CYP2C19, younger age, and being more frequent alcohol consumption in EAC group.

According to the results of some studies from the United States the eradication rate of *H. pylori* by first-line therapy (PPI + Amoxicillin + Clarithromycin) is decreasing in recent years from 75% (Laine, 1998) to 65% (Bochenek, 2003) [17]. As shown from our study the eradication rate in the EAC group was still as high as 91%. As mentioned above, PPIs are metabolized primarily via CYP2C19 pathway. According to the polymorphism of CYP2C19, individuals can be divided into extensive metabolizer (EM) and poor metabolizer (PM). The prevalence of PM is more frequent in Asian population (15–23%) than Caucasian population (2–5%) [24]. The therapeutic effect of PPI in terms of *H. pylori *eradication is better in PM individuals. This observation might explain the higher eradication rate in our study than studies from the United States. In addition, another major determinant for successful eradication is body mass index (BMI). According to Hsu et al. [9] the average body weight of the Asian is less than the Caucasian. Therefore, it is not surprising that higher eradication rate is found Asian populations, if the same dose of proton pump inhibitor and antibiotics are used. 

The interaction between proton pump inhibitor and clopidogrel remains a controversial issue. As recent studies reported PPI and clopidogrel are both metabolized via cytochrome P450 pathway (CYP), especially 2C19 [25]. Therefore, coprescribing PPI and clopidogrel may contribute to decreased cardiovascular protection related to clopidogrel. Esomeprazole is less metabolized than pantoprazole via CYP2C19 pathway [25, 26]. In addition we could administer single-dose esomeprazole in the morning and clopidogrel in the evening or at bedtime during *H. pylori* eradication for reducing the interaction. According to Hsu et al. esomeprazole doesn't have negative effect on clopidogrel about platelet aggregation [27]. Single-dose esomeprazole-based triple therapy is a better option than pantoprazole for patients coprescribed clopidogrel.

In conclusion, our study show that single-dose esomeprazole-based first line triple therapy (esomeprazole 40 mg once daily, amoxicillin 1 g twice daily, clarithromycin 500 mg twice daily) is an effective regimen for *H. pylori* eradication in Taiwan.

## Figures and Tables

**Table 1:** Demographic distribution and Endoscopic diagnosis of two patient groups.

	EAC group (*n* = 208)	PAC group (*n* = 245)	*P* value
Age (years)			
Mean ± SD	50.91 ± 12.96	51 ± 12.34	.016
Gender			.873
Male	89 (42.8%)	103 (42%)	
Female	119 (57.2%)	142 (58%)	
Smoking	27 (13%)	24 (9.8%)	.591
Alcohol consumption	18 (8.7%)	9 (3.7%)	.046
Ingestion of coffee	60 (28.8%)	49 (20%)	.195
Ingestion of tea	85 (40.9%)	79 (32.2%)	.895
Endoscopic diagnosis			.705
Gastritis	60 (28.8%)	82 (33.5%)	
Gastric ulcer	31 (14.9%)	33 (13.5%)	
Duodenal ulcer	94 (45.2%)	101 (41.2%)	
Gastric and duodenal ulcer	23 (11.1%)	29 (11.8%)	

**Table 2:** Outcomes of esomeprazole- and pantoprazole-based triple therapy.

	EAC group (*n* = 208)	PAC group (*n* = 245)	*P* value
Eradication rate			
Intention-to-treat	72% (150/208)	55% (135/245)	<.05
Per-protocol	91% (150/165)	72% (135/187)	<.05
Adverse events	19% (39/208)	17% (42/245)	.712
Compliance	87% (181/208)	91% (223/245)	.083

**Table 3:** Adverse events during single-dosed esomeprazole- and pantoprazole-based triple therapies.

Adverse events	EAC group (*n* = 208)	PAC group (*n* = 244)	*P* value
Diarrhea	7 (3.4%)	10 (4.1%)	.131
Constipation	1 (0.5%)	2 (0.8%)	1.000
Abdominal pain	3 (1.4%)	9 (3.7%)	.241
Anorexia	1 (0.5%)	3 (1.2%)	.633
Nausea	7 (3.4%)	8 (3.3%)	1.000
Vomiting	1 (0.5%)	4 (1.6%)	.388
Skin rash	0 (0%)	5 (2%)	.069
Dizziness	12 (5.8%)	11 (4.5%)	.519
Headache	2 (1%)	8 (3.3%)	.196
Taste perversion	32 (15.4%)	29 (11.9%)	.209
Fatigue	10 (4.8%)	6 (2.5%)	.198

**Table 4:** Clinical factors of higher eradication rate in the study.

Clinical factors	*P*
Age	.016
Alcohol consumption	.046
Prescribed PPI	<.05
